# Novel, Stable Catholyte for Aqueous Organic Redox Flow Batteries: Symmetric Cell Study of Hydroquinones with High Accessible Capacity

**DOI:** 10.3390/molecules26133823

**Published:** 2021-06-23

**Authors:** Xian Yang, Sergio Navarro Garcia, Tobias Janoschka, Dénes Kónya, Martin D. Hager, Ulrich S. Schubert

**Affiliations:** 1 Laboratory of Organic and Macromolecular Chemistry (IOMC), Friedrich Schiller University Jena, Humboldtstrasse 10, 07743 Jena, Germany; xian.yang@jenabatteries.de (X.Y.); martin.hager@uni-jena.de (M.D.H.); 2 Center for Energy and Environmental Chemistry Jena (CEEC Jena), Friedrich Schiller University Jena, Philosophenweg 7a, 07743 Jena, Germany; 3 JenaBatteries GmbH, Otto-Schott-Strasse 15, 07745 Jena, Germany; tobias.janoschka@jenabatterie.de; 4 Research Centre for Natural Sciences, Magyar Tudosok Korutja 2, 1117 Budapest, Hungary; sergio.navarro@ttk.hu

**Keywords:** hydroquinone, catholyte, stability, symmetric cell study, redox flow batteries, energy storage

## Abstract

Owing to their broad range of redox potential, quinones/hydroquinones can be utilized for energy storage in redox flow batteries. In terms of stability, organic catholytes are more challenging than anolytes. The two-electron transfer feature adds value when building all-quinone flow battery systems. However, the dimerization of quinones/hydroquinones usually makes it difficult to achieve a full two-electron transfer in practical redox flow battery applications. In this work, we designed and synthesized four new hydroquinone derivatives bearing morpholinomethylene and/or methyl groups in different positions on the benzene ring to probe molecular stability upon battery cycling. The redox potential of the four molecules were investigated, followed by long-term stability tests using different supporting electrolytes and cell cycling methods in a symmetric flow cell. The derivative with two unoccupied ortho positions was found highly unstable, the cell of which exhibited a capacity decay rate of ~50% per day. Fully substituted hydroquinones turned out to be more stable. In particular, 2,6-dimethyl-3,5-bis(morpholinomethylene)benzene-1,4-diol (**asym-O-5**) displayed a capacity decay of only 0.45%/day with four-week potentiostatic cycling at 0.1 M in 1 M H_3_PO_4_. In addition, the three fully substituted hydroquinones displayed good accessible capacity of over 82%, much higher than those of conventional quinone derivatives.

## 1. Introduction

Research on redox flow batteries (RFBs) has, in recent years, shifted from metal-based materials to organic active materials that are abundant in nature and have great potential to reduce costs by improving synthetic strategies [1,2]. Since the redox-active organic molecule tiron (4,5-dibenzoquinone-1,3-benzenedisulfonate) was initially introduced as a catholyte in RFB in 2009 [3], it has aroused extensive attention in developing novel, redox-active organic molecules with high solubility and stability for practical redox flow battery applications. To date, quite a few candidates have been investigated in RFBs [4,5,6,7,8,9,10,11,12,13,14], among which two organic RFB systems, the TEMP-TMA/MV (*N*,*N*,*N*-2,2,6,6-heptamethylpiperidinyl oxy-4-ammonium chloride/*N*,*N*′-dimethyl-4,4-bipyridinium dichloride) system and ferrocyanide/anthraquinone system, were validated and industrialized in the first place [13,14]. The TEMP-TMA/MV system achieved a capacity of 54 Ah·L^−1^ at a concentration of 2 mol·L^−1^, displaying a total energy density of 38 Wh·L^−1^ at a cell voltage of 1.4 V [14]. The ferrocyanide/anthraquinone system was cycled at a constant current density of 100 mA·cm^−2^ for 100 charging/discharging cycles, resulting in a coulombic efficiency of over 99% with a stable round-trip efficiency of 84% and 0.1%/cycle loss of capacity, which was expected to be negligible when the system size was scaled up [13]. Yet the most challenging issue for the development of organic redox flow batteries (ORFBs) remains relatively low energy density. Possible approaches to this challenge lie in broadening the electrochemical window (ECW), enhancing the solubility and/or redox potential of the redox-active materials [5]. Non-aqueous solvents have a rather expanded ECW varying from −4 to 2.3 V (vs. ferrocene/ferrocenium) [15]. In contrast, the ECW of aqueous ORFBs is greatly constrained by the water stability window (~1.23 V). Despite the superiority of the wide ECW of non-aqueous ORFBs, they face several challenges including flammability, poor solubility of the redox pairs in organic solvents, high viscosity and low conductivity of electrolyte solutions as well as a special membrane design which is required to resist against swelling by organic solvents [16]. Comparatively, aqueous ORFBs have the merits of a low-cost solvent (water) and supporting electrolytes (basically, inorganic salts), high ion conductivity, fast kinetics and, most importantly, long-term safety for practical applications [1,17]. As the redox potential varies with the pH value of the electrolyte solution, it is possible to enlarge the ECW of water to some extent. There are also examples of applying highly concentrated electrolytes to broaden the voltage window of the aqueous ORFBs [15,18]. Although there have been limited improvements to enlarging the cell voltage of aqueous ORFBs, it has indeed been possible to design new redox pairs with high solubility to fit into the water stability window. Meanwhile, with the help of theoretical computing methods such as density-functional theory (DFT), researchers have been able to screen for promising candidates from numerous alternatives, which will accelerate the development of new redox pairs [19,20]. Another notable way of raising energy density lies in increasing the number of electrons stored per molecule, e.g., using quinones instead of TEMPO radicals.

Organic molecules are flexible regarding tailoring their properties. For instance, positively or negatively charged ions can be easily incorporated into parent molecules to be well compatible with anion or cation exchange membranes, meanwhile enhancing solubility in aqueous solutions. The redox potential of organic molecules can be shifted within a certain range by introducing electron-withdrawing groups (EWGs) or electron-donating groups (EDGs) [21,22]. As promising candidates, quinone/hydroquinone derivatives have advantages over other types of redox-active organic molecules. They deliver two electrons for each half-cell reaction and can serve as both catholytes and anolytes, which is an intriguing point regarding building metal-free all-quinone based ORFBs [21]. In a previous work, 28 quinone derivatives were investigated systematically in aqueous solutions using cyclic voltammetry (CV) and other techniques [4]. 2,6-anthraquinone disulfonic acid (2,6-AQDS), 2,7-anthraquinone disulfonic acid (2,7-AQDS) and their derivatives can be employed as anolytes in aqueous RFBs, whereas hydroquinones (HQs) have sufficiently positive redox potential to be used as catholytes. Although AQDS is known for its low cost, high solubility, chemical stability and rapid redox kinetics [23], it forms electrochemically inactive dimers at the concentrations employed for practical applications, which indirectly decreases the energy storage capacity [24,25,26]. Most known HQs with high redox potential are not sufficiently stable for flow battery applications [4]. Hence, there is an urgent need to develop stable and highly soluble HQs for the use in ORFBs, in particular for the positive electrode of the battery, i.e., catholyte solution. In hydroquinone form, all of the unsubstituted carbon atoms in the aromatic ring are prone to electrophilic substitution and nucleophile substitution for the quinone form, i.e., the Michael addition reaction [27,28]. Our supposition is that if these unsubstituted carbon atoms are blocked, this could lead to more stable molecules. It has further been revealed that the 1,2- and 1,4-HQs represent promising candidates as catholytes for ORFBs, wherein the fully substituted HQs could gain greatly enhanced solubility in aqueous solutions [4]. Moreover, fully substituted HQs are supposed to mitigate side reactions, either by blocking radical transfer via the tautomerization of the relatively acidic aromatic protons, or by the introduction of steric, kinetic hindrance using bulky functional groups such as morpholinomethylene [29]. To validate the hypothesis, a HQ fully substituted by tetramorpholinomethylene groups (TMHQ) was synthesized and tested at a concentration of 0.1 M in 1 M NH_4_Cl in a symmetric flow cell set-up [30]. In acidic conditions, the morpholinomethylene group was protonated to obtain a quaternary ammonium group, turning the EDG into an EWG. Consequently, the redox potential was shifted to 0.89 V. The molecule featured a high solubility (2 M in 37% hydrochloric acid). However, the capacity retention over 100 charging/discharging cycles was only 33%, with a potentiostatic cycling between −0.3 and 0.3 V, far below expectations. Hence, a deeper understanding of the stability of HQs is necessary for guiding the future design of HQ derivatives possessing both high solubility and stability.

Therefore, a series of four HQ derivatives incorporating the morpholinomethylene and/or methyl groups were designed to understand the relationship between the structure of this particular compound class and the important parameters of a redox flow battery. The influence of molecular structure on the solubility of the four molecules were thoroughly discussed in our previous report [31]. In this work, the stability of the four molecules was investigated in symmetric flow cells in different supporting electrolytes using different cycling methods. Among them, 2,5-bis(morpholinomethylene)benzene-1,4-diol (**O-3**) was substituted with two morpholinomethylene groups in symmetric positions without protecting the other two ortho positions. As a comparison, 2,5-dimethyl-3,6-bis(morpholinomethylene)benzene-1,4-diol (**O-5**) was fully substituted on the basis of **O-3**, where the unprotected *ortho* positions were occupied by two methyl groups. 2,6-dimethyl-3,5-bis(morpholinomethylene)benzene-1,4-diol (**asym-O-5**) possessed the same chemical composition as **O-5** but with molecular central asymmetry. The fourth molecule was 2,3,5-trimethyl-6-(morpholinomethylene)benzene-1,4-diol (**O-1**), which was fully substituted with one morpholinomethylene group and three methyl groups. The capacity decay rate of symmetric cells revealed the stability of the given molecule. Our goal was to check the importance of protecting the *ortho* positions of hydroquinones and to prove our supposition that fully substituted hydroquinones are stable and exhibit high accessible capacity, beneficial for flow battery applications.

## 2. Results

### 2.1. Reversibility and Redox Potential

**Figure 1:**
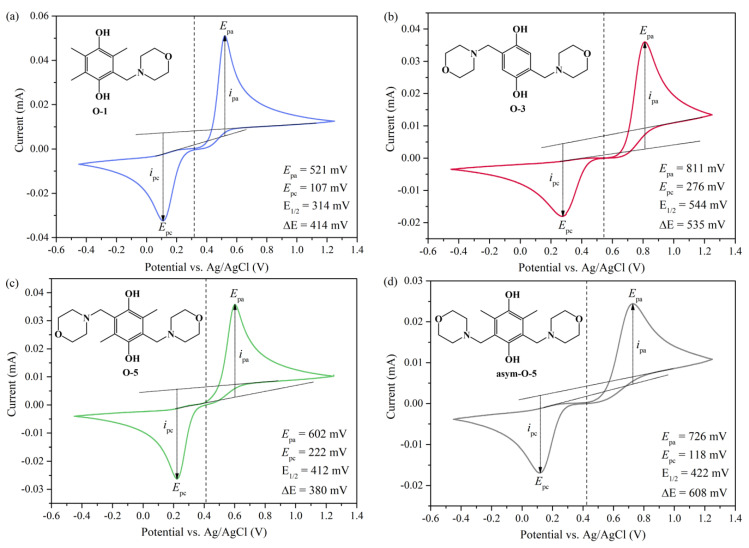
Cyclic voltammographs of hydroquinones (**a**) **O-1**, (**b**) **O-3**, (**c**) **O-5** and (**d**) **asym-O-5** measured at a scan rate of 100 mV·s^−1^ in 1 M H_2_SO_4_. The chemical structures of the four molecules are presented in the top-left corner of the corresponding subfigures. The dashed line indicates the half-wave potential E_1/2_ vs. Ag/AgCl.

The electrochemical reversibility of the four molecules was investigated via cyclic voltammetry (CV), and the redox potentials were obtained, as shown in Figure 1. The oxidation of hydroquinones involved a two-electron transfer process coupled with proton transfer. The cathodic peak current (*i*_pc_) and the anodic peak current (*i*_pa_) could provide information on the reversibility of the redox-active molecules. When *i*_pa_/*i*_pc_ equaled or was very close to one, it indicated the reversibility of the investigated molecules such as in the cases of **O-1**, **O-5** and **asym-O-5** (Figure 1a–d). When *i*_pa_/*i*_pc_ was far from one, for example, the value for **O-3** was 1.5 (Figure 1b), and it was irreversible. In a two-electron transfer half-cell reaction, the ideal peak separation ΔE_p_ was 29.5 mV for an electrochemically reversible process. The peak separation for the four molecules ranged from 380 to 608 mV, way beyond the theoretical expectation. This phenomenon was assigned to the low kinetics on the glassy carbon electrode [32].

Upon being dissolved in H_2_SO_4_, the morpholinomethylene group changed from an EDG to an EWG, as it was protonated to a quaternary ammonium cation, enhancing the solubility as well as leading to redox potential shift toward positive values. As an EDG, the methyl group decreased the redox potential. Consequently, the lowest redox potential of 0.54 V was assigned to **O-1,** which bore three electron-donating methyl groups and one electron-withdrawing morpholinomethylene group. In contrast, the redox potential of **O-5** was more positive (0.64 V) because it had one less methyl group and one more morpholinomethylene group than **O-1**. **Asym-O-5** had the same substituent groups but in different positions on the benzene ring, resulting in a redox potential (0.65 V) close to that of **O-5**. With no methyl group present, **O-3** revealed the highest redox potential of 0.77 V.

### 2.2. Symmetric Cell Cycling Tests

**Scheme 1:**
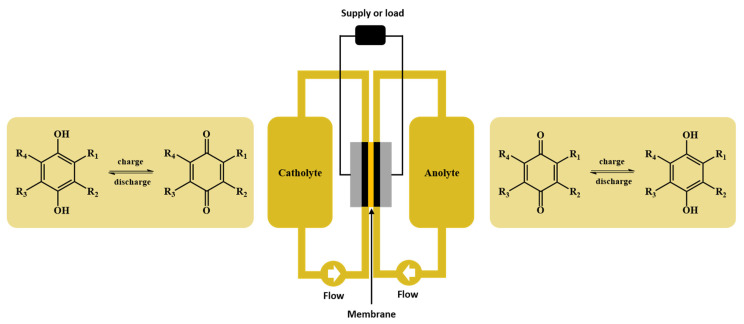
Schematic representation of the redox flow battery setup and the redox reactions occurring upon charging and discharging of the battery.

In symmetric cell cycling tests, identical solutions of the substance to be analyzed were employed for anolytes and catholytes (Scheme 1). Starting from a state of charge (SOCs) of 50%, the battery was charged and discharged repeatedly, and the capacity was monitored.

Generally, in a full cell setup, the capacity fade involves several mechanisms, mainly stemming from electrolyte degradation, membrane crossover and side reactions. In a symmetric cell, however, as exactly the same electrolyte composition is applied on both sides of the cell, capacity fade due to cross-contamination of the redox-active species can be excluded. Therefore, the capacity decay of the cell over time indicates the chemical stability of the investigated molecules, i.e., the stability of redox-active molecules can be quantified as the capacity decay per unit time [33,34].

#### 2.2.1. Stability

The stability of the four hydroquinone derivatives was primarily investigated in a symmetric cell setup at a concentration of 0.1 M in 1 M H_3_PO_4_, a solvent in which all four molecules were well soluble (0.48 M for **O-1**, 0.53 M for **O-3**, 0.40 M for **O-5** and 0.55 M for **asym-O-5**) [31]. An anion-exchange membrane was employed to retain the protonated, cationic hydroquinones and conduct the counter ions at the same time.

In this kind of test, two cycling methods are commonly employed, the potentiostatic (PS) and the galvanostatic (GS) cycling method. PS cycling is more encouraged than GS cycling, in particular for molecules with low decomposition rates [34]. It is less vulnerable to changes in cell resistance and can access higher shares of the theoretical capacity than the GS cycling method [33]. As H_3_PO_4_ is a weak electrolyte, its solution conductivity is lower than H_2_SO_4_ at the same concentration. Therefore, the PS cycling method was employed as the standard method in this work.

**Figure 2:**
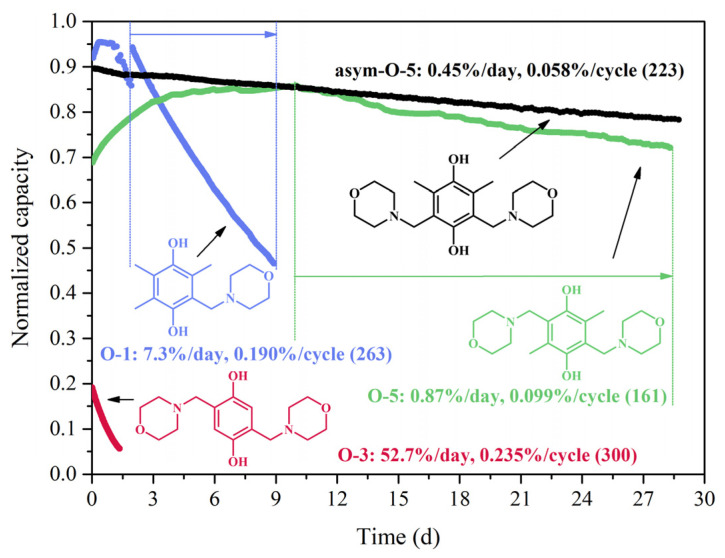
Discharge capacity of the symmetric cells of 0.1 M **O-1**, **O-3**, **O-5** and **asym-O-5** in 1 M H_3_PO_4_ using the PS cycling method. The *y*-axis is normalized by dividing the discharge capacity value by its theoretical capacity. The potential is set at 0.3/−0.3 V for charge/discharge cycles with cut-off current at 3/−3 mA.

The capacity fade of the four hydroquinones over time after being charged/discharged at a constant potential of 0.3/−0.3 V for over 300 cycles is shown in Figure 2. They displayed distinctly different capacity decay rates in 1 M H_3_PO_4_. **O-3**, bearing only two substituents on the hydroquinone core and two unprotected *ortho* positions, showed the lowest stability with a capacity drop of 71% in less than two days. The cell of **O-1** exhibited a moderately fast decay at 7.3% per day and 0.190% per cycle for 263 cycles. **O-5** displayed a decay of 0.87%/day and 0.099%/cycle calculated from its peak capacity (day 10). After four weeks of cycling, the final capacity of the cell with **O-5** remained at 70% of its theoretical value. As a comparison, the capacity decay of **asym-O-5** was 0.45%/day and 0.058%/cycle over four-week cycling for 223 cycles. Even though the capacity decay curves of **O-1** and **O-5** were not smooth at the beginning, one could easily identify **asym-O-5** as the most stable among the four molecules, whereas **O-3** was the least stable. This observation provides evidence for the claim that the ortho positions of hydroquinones need to be occupied to ensure their chemical stability in aqueous solutions [4].

**Figure 3:**
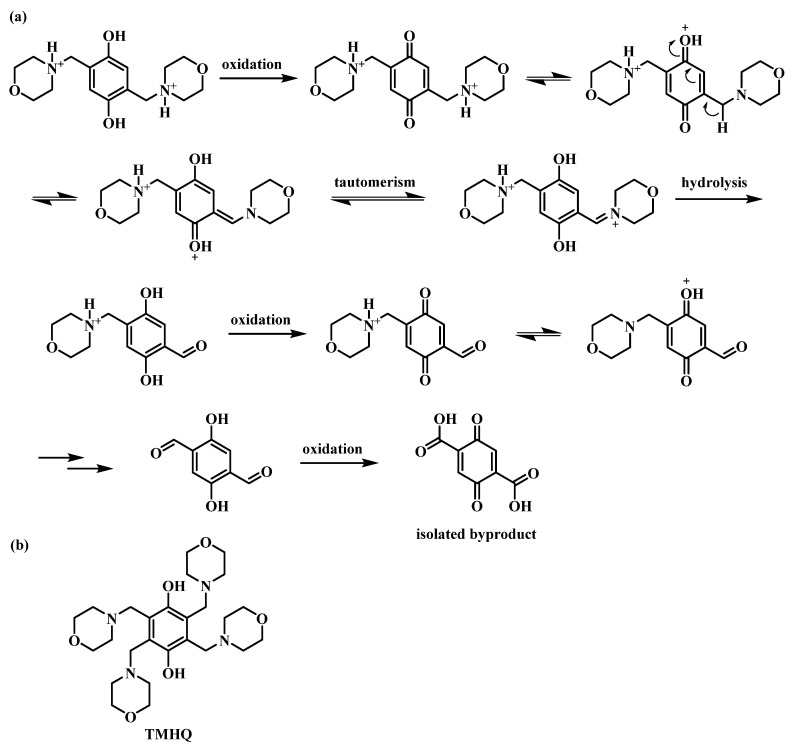
(**a**) Schematic representation of the proposed decomposition pathway of **O-3**; (**b**) schematic representation of the chemical structure of TMHQ.

After cycling, the electrolyte solution of **O-3** was investigated by liquid chromatography—mass spectroscopy (LC–MS), and 2,5-dioxoterephthalic acid was identified as the decomposition product. The derived decomposition pathway of **O-3** (Figure 3a) was found to be similar to that of TMHQ as reported in a previous work [30]. As both the 2,5-dihydroxyterephthalic acid and its quinonic analogue were poorly soluble in water, a white precipitate was found on the surface of the carbon-felt electrode after the flow cell was dismantled. The chemical identity of the by-product was further confirmed by comparing the chromatographic and spectroscopic (UV and MS) properties of the electrochemically oxidized, commercially available dihydroxy terephthalic acid, which were found to be identic.

In summary, the high instability of **O-3** necessitated the protection of all four *ortho* positions of 1,4-HQ. Among the three fully substituted hydroquinones, the substitution of two morpholinomethylene and two methyl groups (**O-5** and **asym-O-5**) was more stable than that of one morpholinomethylene and three methyl groups (**O-1**). It is noteworthy that **O-3** had a rather low initial normalized capacity (~20%) compared to the other three molecules (above 70%). This difference was assigned to the rapid capacity decay rate of **O-3** (rf. Section 2.2.3 for details).

#### 2.2.2. PS vs. GS Cycling Method

As mentioned previously, the preferred method to determine the stability of investigated molecules through a symmetric cell cycling test is the PS method, as it eliminates the influence of the changing cell resistance amid the cycling process [33]. In practical applications, the charge/discharge process may include both PS and GS steps. Therefore, the GS cycling method was studied as an additional way to look at the stability of **O-1**, **O-3**, **O-5** and **asym-O-5**, thereby comparing it with the PS cycling method (Table 1). As H_3_PO_4_ was less conductive, and the GS cycling method was vulnerable to changing cell resistance, H_2_SO_4_ was chosen as the supporting electrolyte except in **O-3**, which was poorly soluble in H_2_SO_4_.

**Figure 4:**
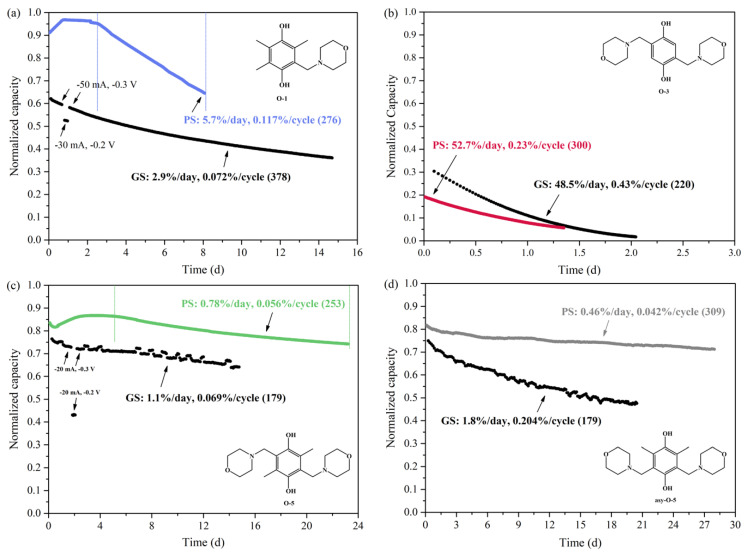
Discharge capacity of the symmetric cell tests of (**a**) **O-1**, (**b**) **O-3**, (**c**) **O-5** and (**d**) **asym-O-5** using the PS and GS cycling methods. The *y*-axis is normalized by dividing the discharge capacity value by its theoretical capacity. The potential was set at 0.3/−0.3 V with cut-off current at 3/−3 mA for the PS cycling method. The charge/discharge current applied in the GS cycling method was 50/-50 mA for **O-1**, 30/−30 mA for **O-3** and 20/−20 mA for **O-5** and **asym-O-5** with a cut-off voltage of 0.3/−0.3 V.

Obviously, for all four molecules, a significant difference in capacity decay rate was found when using different cycling methods. When the PS cycling method was applied, the overall stability trend stayed the same in H_2_SO_4_ as in H_3_PO_4_, i.e., **asym-O-5** > **O-5** > **O-1** > **O-3** (Figure 4). However, when the molecules were charged/discharged at a constant current, the capacity decay of the four molecules displayed non-ignorable difference from the PS cycling method. In particular, **O-5** exhibited the lowest capacity decay rate, presenting higher stability than **asym-O-5**. It should be noted that several parameters could influence the observed capacity decay rate, e.g., reactant concentration and state of charge (SOC). On the one hand, higher reactant concentration (starting from 0.1 M) has been observed in the literature to result in lower capacity retention, which relates mainly to the reactant’s stability [35]. On the other hand, it was reported that 0.1 M 2,6-DHAQ in 1 M KOH experienced much faster capacity decay (around 10%/day) at 100% SOC than at 50% SOC (around 1%/day) [33]. For 0.1 M 2,7-AQDS in 1 M H_2_SO_4_, the average capacity decay was found to be 0.08 ± 0.02%/day. The highest capacity observed was 0.2%/day at 100% SOC [33]. To confirm whether this observation was applicable to hydroquinones, the SOC of **O-1** was changed by altering the cut-off voltage and charging current. When the charge/discharge current of **O-1** was 50 mA, 62.5% of the theoretical capacity was achieved before it reached the cut-off voltage. When the charge–discharge current decreased to 30 mA and the cut-off voltage to 0.2/−0.2 V, the accessible capacity decreased, and the capacity decay dropped accordingly. Therefore, the lower initial concentration and SOC collectively led to the lower capacity decay rate of **O-1** when using the GS cycling method (Figure 4a). Similarly, the SOC of **O-5** was reduced by lowering the applied potential, and the corresponding rate of capacity decay decreased (Figure 4c). It is interesting that **O-5** and **asym-O-5** stood at similar starting points upon charging and discharging, but the capacity of the cell with **asym-O-5** decayed faster compared to **O-5** at a constant discharging potential. This might be assigned to the influence of reactant concentration (Table 1). Similar to using the PS cycling method, due to its fast decomposition, **O-3** revealed a high-capacity decay rate when using the GS cycling method at 0.1 M concentration in 1 M H_3_PO_4_ (Figure 3). The capacity was almost used up within two days. Nevertheless, it is worth noting that the GS cell could access a higher share of the theoretical capacity in its initial cycles at a charge/discharge current of 30/−30 mA (Figure 4b).

**Figure A1:**
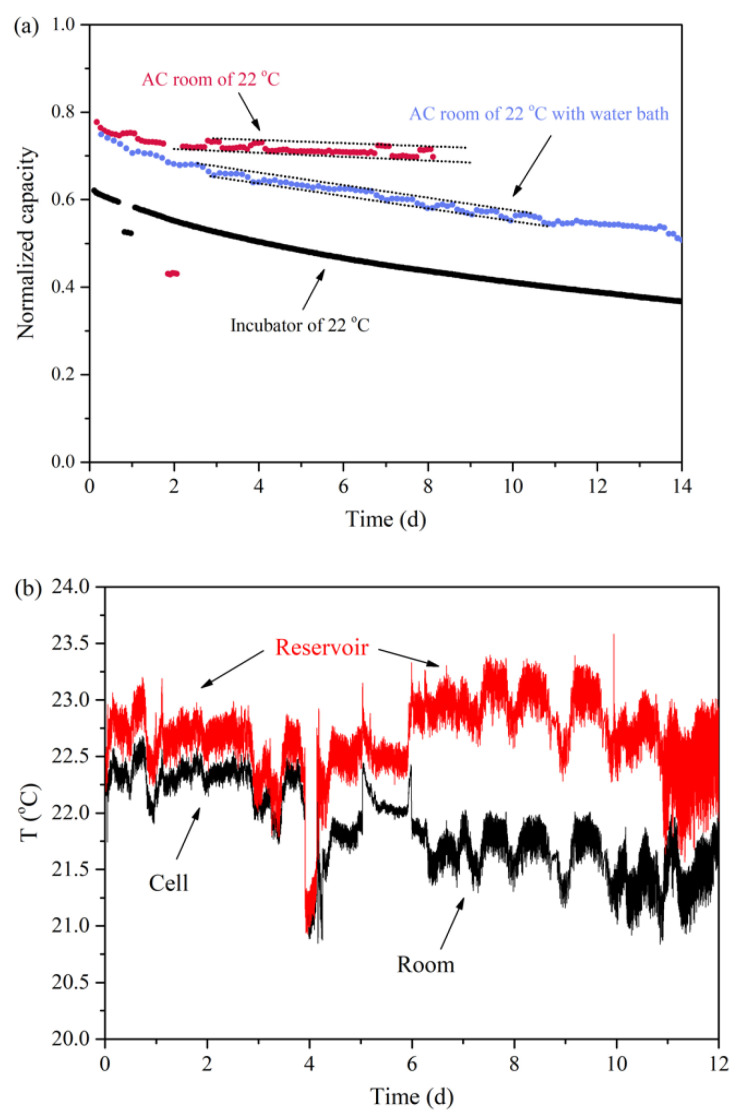
(**a**) Discharge capacity of a symmetric flow cell using the GS cycling method under different temperature control conditions; (**b**) temperature of the air-conditioned (AC) room, the cell setup and the electrolyte reservoir.

Furthermore, apparent capacity fluctuations were observed for both **O-5** and **asym-O-5,** using the GS cycling method, but not for **O-1** and **O-3**. That was because the cell of **O-1** was placed in a temperature-controlled chamber, while the cells of **O-5** and **asym-O-5** were operated at room temperature (Figure A1 in Appendix A). In the case of **O-3**, the decomposition happened too fast to reflect upon the curve. Although temperature variation did not lead to direct capacity fade, it provided evidence for the need to use the PS cycling method to obtain a smooth trend for unfolding the actual stability of the investigated molecules.

#### 2.2.3. Accessible Capacity

Redox-active molecules with good solubility and stability represent promising materials for practical flow battery application. Their electrolyte utilization is also an important factor in order to achieve high accessible capacity, meaning the ratio of practically observed discharge capacity over its theoretical value as given by the concentration of the redox-active molecule and the solution volume. Table 2 summarizes the accessible capacity of the four molecules in 1 M H_3_PO_4_ and H_2_SO_4_, acquired using the PS cycling method. **O-3** showed the least accessible capacity (20%), **O-5** and **asym-O-5** ranked in the middle and the cell of **O-1** utilized almost its full capacity (96% in H_3_PO_4_ and 97% in H_2_SO_4_).

Apparently, all four hydroquinones derivatives were unable to use the full theoretical capacity right from the beginning. This effect is known from the literature, as many quinone-based systems suffer from poor accessible charge capacity due to the dimerization of the redox-active species [25]. There are two kinds of dimerization. One is the formation of dimers upon dissolving the substance in a solvent. The other one arises from the association of the hydroquinone with its oxidized quinone form, i.e., quinhydrone formation [24]. As a result, the two-electron transfer in one half-reaction is usually hindered at concentrations commonly employed in practical flow batteries.

**Figure 5:**
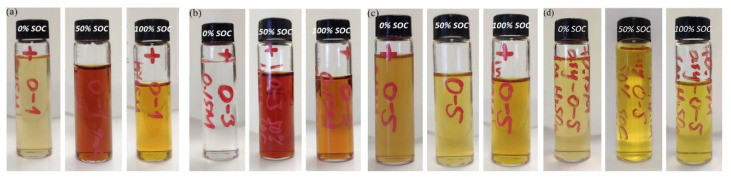
Colors of hydroquinone solutions at different SOCs, indicating the charge/discharge status of the flow cell. (**a**) 0.15 M **O-1** in 1 M H_2_SO_4_ at 0% (light yellow), 50% (red) and 100% (orange) SOC; (**b**) 0.15 M **O-3** in 1 M H_3_PO_4_ at 0% (colorless), 50% (red) and 100% (orange red) SOC; (**c**) 0.1 M **O-5** in 1 M H_2_SO_4_ at 0% (orange red), 50% (light yellow) and 100% (bright yellow) SOC; (**d**) 0.15 M **asym-O-5** in 1 M H_2_SO_4_ at 0% (light yellow), 50% (yellow) and 100% (bright yellow) SOC.

One way to recognize the formation of quinhydrone is the apparent color change of the solution once charged in the flow cell. For instance, electrochemical studies on anthraquinone derivatives have shown that the formation of a hydroxyanthraquinone–anthraquinone dimer leads to an apparent solution color change from yellow/colorless to green [24,36]. The color change of **O-1**, **O-3**, **O-5** and **asym-O-5** solutions over different SOCs suggested that the investigated hydroquinones formed quinhydrones (Figure 5).

**Figure A2:**
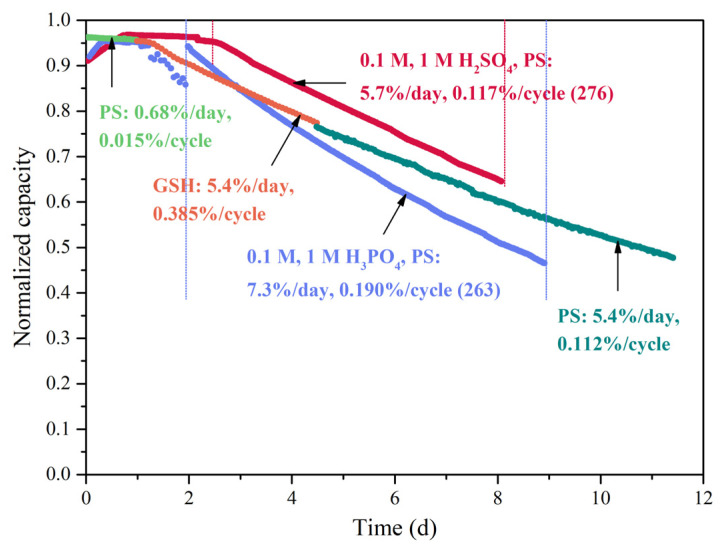
Capacity decay rate of **O-1** in different supporting electrolytes (H_3_PO_4_ or H_2_SO_4_), using PS and GSH (galvanostatic with potential hold) cycling methods.

Looking at the data of Figure 2 in detail, **O-1** accessed 92% of its theoretical capacity in the first charge/discharge cycle. It then directly climbed up to 96% in a half-day period and held for two days before it started to decline. For comparison, in the symmetric cell cycling test of TMHQ using the PS cycling method, a similar plateau at the starting point of the capacity decay curve was found in a literature example, where it held at 92% of its theoretical capacity for five cycles before its fast decay [30]. In another work, Hagemann investigated the cycling stability of a new, polymer-based catholyte using the GS cycling method, and no plateau was observed [8]. Hence, the existence of this plateau may be related to the cycling method. This observation was supported by studies of **O-1**, **O-3**, **O-5** and **asym-O-5** where no capacity-holding period was observed when applying the GS cycling method (Figure 4). In addition, symmetric cell cycling tests of 0.1 M **O-1** in different supporting electrolytes, applying different cycling methods were performed as further evidence to explain this plateau phenomenon (Figure A2 in Appendix A).

The charge/discharge capacity of **O-5** experienced a slow increase in the first ten days and then underwent a constant decay. Likewise, this phenomenon was related to the cycling method applied. In an independent experiment, the symmetric cell cycling tests of **O-5** were performed in different supporting electrolytes at a concentration of 0.1 M while using the same PS cycling method. The period of capacity increase from the beginning was apparent in both 1 M H_2_SO_4_ and H_3_PO_4_ (Figure 2 and Figure 4c, green curves). Peak values were at about 85% and 88% of theoretical capacity. In contrast, the cell for preparation of the 100% SOC solution of **O-5** against vanadium(III) successfully achieved 99.8% of the theoretical capacity, indicating the material’s capability to access full capacity at a concentration of 0.1 M and suggesting that the formation of dimers from two single molecules of **O-5** was reversible. Surprisingly, the cell of **asym-O-5**, for which the electrolyte reservoirs were cooled by water bath, did not undergo a long period of pre-conditioning before the capacity started to decay. This suggests that the underlying reason for the capacity increase in the cases of **O-1** and **O-5** might be temperature changes when applying the PS cycling method, which is supposed to affect the complicated dimerization process.

The first cycle of **O-3** utilized only 20% of the theoretical capacity. In contrast, during the preparation of the 100% SOC solution, the cell reached 99.4% of the theoretical capacity, which meant that if dimers were formed in the solution, the reaction should have been highly reversible. It was possible that the dimerization constant for quinhydrone formation was large enough to hinder the reverse reaction, as 100% SOC solution of **O-3** was prepared successfully against V(III). Under the same conditions, **O-1** and **O-5** both displayed a period of capacity increase before the capacity started to drop. As this was not observed in the case of **O-3**, it was safe to assume the decomposition of **O-3** started to occur as soon as the hydroquinone was fully oxidized during the preparation of the symmetric cell test setup.

## 3. Discussion

The symmetric cell cycling tests of the four hydroquinones unfold their stability in a good way and provide solid evidence for the importance of fully protecting the ortho positions of HQs. In 2018, Goulet et al. published a systematic study determining the stability of 2,7-AQDS, 2,6-DHAQ, MV and bis(trimethylammoniopropyl)viologen (BTMAPV) at a concentration of 0.1 M [33]. The two quinone derivatives, 2,7-AQDS and DHAQ, exhibited a capacity decay rate of 0.08~0.2%/day and 1~10%/day, respectively, depending on the SOC. The viologen derivative MV showed a capacity decay rate of 1.5%/day, whereas cycling of BTMAPV (bis(trimethylammoniopropyl)viologen) displayed negligible capacity loss (within measuring error) over a two-week operation. Referring to the classification by Aziz et al. the capacity decay rate of >1%/day is categorized to be ‘high’, 0.1~1%/day is regarded as ‘moderate’ and <0.02%/day is ‘low’ [34]. In this work, the most stable **asym-O-5** had a capacity decay rate of 0.45%/day (Table 3) at 0.1 M in 1 M H_3_PO_4_, even upon cycling to a high SOC value (90%). The moderately low capacity decay rate of **asym-O-5** demonstrated that it was a reliable, redox-active hydroquinone alternative that could be used as a catholyte and suggested the benefit of substituting all benzylic protons. As an alternative candidate for flow battery application, **O-5** had a capacity decay rate of 0.78 to 1.1%/day depending on the supporting electrolytes, cycling method and concentration. However, it should be noted that solubility of **O-5** in H_2_SO_4_ changed from 0.24 M to 0.06 M during storage over time. This effect stemmed from the formation of a less-soluble polymorph induced by molecular central symmetry [31]. In comparison, **asym-O-5**, bearing identical substituents as **O-5** but a central asymmetry, maintained its solubility of 0.44 M in pH 1 H_2_SO_4_ and 0.55 M in pH 1 H_3_PO_4_.

As a popular selection for the anolyte, AQDS suffers from poor accessible capacity due to dimerization in non-dilute solutions [25]. As shown in Table 4, the combination of 0.1 M BQDS and 0.1 M 2,7-AQDS in 1 M H_2_SO_4_ made 65% of theoretical capacity practically available in a flow cell. When the concentration was increased to 1 M, the accessible capacity decreased to 26%, as the equilibrium shifted towards dimer formation. At the same concentration, a cell employing BQDS and 2,6-AQDS (1 M in 1 M H_2_SO_4_) revealed an accessible capacity of 35%. A third example illustrating the problem at the low concentration of 0.05 M in 1 M H_2_SO_4_ was a flow cell using BQDS and ARS. It reached less than half of the theoretical capacity despite the low concentration.

**Figure A3:**
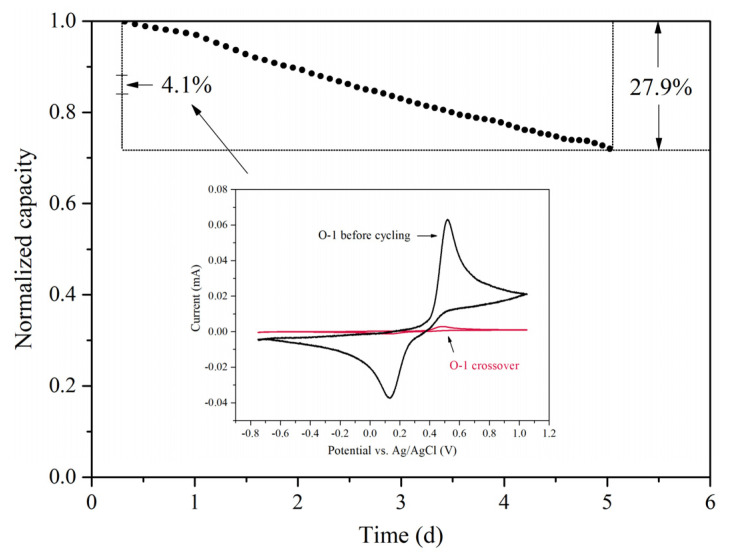
Discharge capacity fade over time. The cell employed 0.5 M **O-1** in 1 M H_2_SO_4_ as the catholyte and 1.6 M V(III) as the anolyte. Charge/discharge current was set at 200 mA. The inserted figure is the cyclic voltammogram of **O-1** before cycling and its crossover to the anode side after cycling.

In this work, except for **O-3,** which was highly unstable, three molecules exhibited high accessible capacity (Table 2, 82~97%) in the symmetric cell test, an advantage for redox flow battery applications. In particular, **O-1** could achieve 96% of its theoretical capacity in H_3_PO_4_ and 97% in H_2_SO_4_ at 0.1 M, which was maintained even when the concentration increased to 0.5 M in a full cell test with V(III) as the anolyte (Figure A3 in Appendix A).

## 4. Materials and Methods

### 4.1. Synthesis, Analytical Description and Solubility Measurements

The synthetic methods of the compounds ^1^H NMR, LC-MS as well as solubility data can be found in our previous report [31].

### 4.2. Cyclic Voltammetry

Cyclic voltammetry was employed to determine the redox potential of the four hydroquinones. All cyclic voltammograms were recorded using a VMP3 potentiostat (BioLogic, France) via a three-electrode cell, where a glassy carbon served as the working electrode, platinum wire as the counter electrode and silver/silver chloride as the reference electrode. A representative measurement can be described as follows:

The cell was first filled with 5 mL 1 M H_2_SO_4_ aqueous solution and then de-aerated with argon for 30 s. The cyclic voltammograms were recorded within the water stability window from −0.45 to 1.25 V at a scan rate of 100 mV·s^−1^. Six consecutive scans were applied to acquire credible results. Subsequently, 10 mg sample was added to the reservoir, which was then de-aerated again and measured following the aforementioned protocol. After subtracting the background, the fourth scan was taken as the reference for calculating the redox potentials of the investigated molecules.

### 4.3. Preparation of Electrolyte Solutions

All cells mentioned in this work were based on a lab-scale single cell with an active area of 5 cm^2^ (JenaBatteries GmbH, Jena, Germany). The cell setup used was adapted from what was described previously [9].

Vanadium (III) preparation: The V(III) electrolyte was prepared from the 1.6 M commercial V(IV)/V(III) mixed solution (GfE Metalle und Materialien GmbH) with N117 (Ion Power GmbH) as the separator. It was charged at 20 mA until it reached the cut-off voltage of 1.25 V or its theoretical capacity. The solution obtained on the anode side (V(III)) was then used to prepare the 100% state of charge (SOC) solutions of **O-1**, **O-3** and **O-5**.

Preparation of 50% SOC solutions: The 50% SOC solutions of **O-1**, **O-3** and **O-5** were prepared by mixing the same volume of the corresponding 100% SOC solution with the 0% SOC solution of the same concentration. The 100% SOC solutions of **O-1**, **O-3** and **O-5** were obtained by charging them against the aforementioned V(III) solution. By applying an anion exchange membrane (FAPQ-350, Fumatech GmbH, Bietigheim-Bissingen, Germany), the crossover of vanadium species could be avoided, ensuring the purity of the obtained solution. The 0% SOC solutions of **O-1**, **O-3** and **O-5** were made from dissolving a certain amount of solid in 1 M H_2_SO_4_ or H_3_PO_4_. The cells were charged at an appropriate current (20 to 50 mA, depending on theoretical capacity and cell resistance) to the cut-off voltage of 1.3 V, marking the achievement of full capacity, i.e., 100% SOC solution was successfully obtained.

### 4.4. Symmetric Cell Cycling Tests

**Figure 6:**
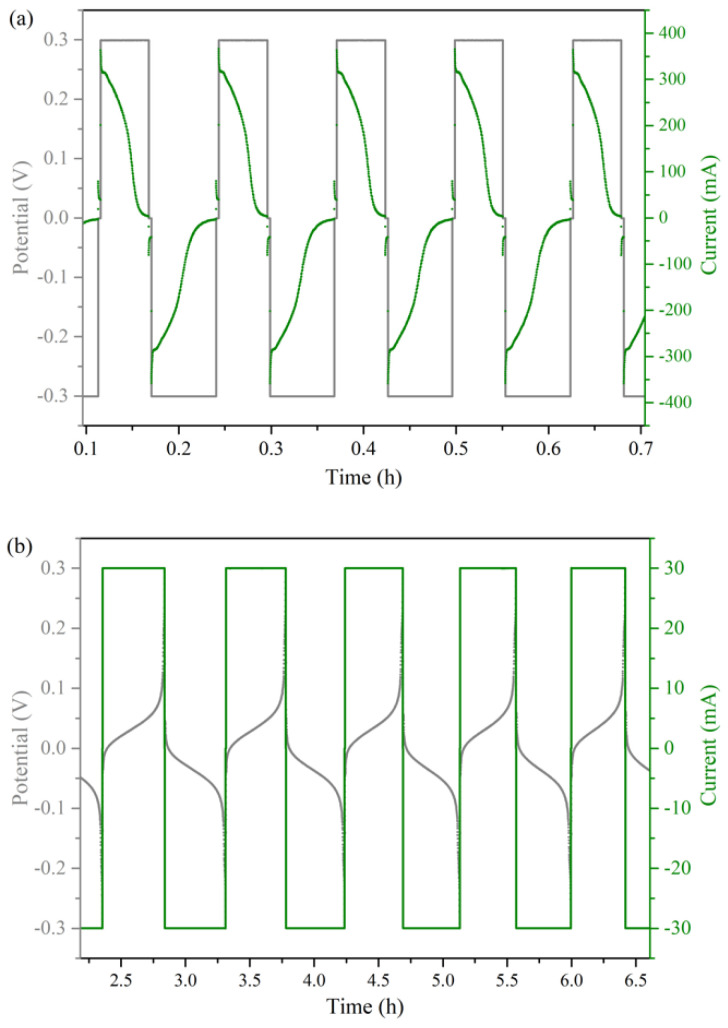
Examples of (**a**) the PS cycling method and (**b**) the GS cycling method.

Symmetric cell testing was first introduced in 2013 to reduce crossover [41]. It was later demonstrated by Milshtein et al. as a method to study the capacity fade of a single electrolyte [42,43]. In 2018, Goulet et al. developed this method further by defining the capacity limiting side (CLS), linking the capacity fade of the cell directly to the stability of the investigated molecule [33]. It soon became a validated and efficient tool to screen new redox active species for their potential application in redox flow batteries. Starting from the 50% SOC electrolyte solutions on both sides of the cell, the capacity decay caused by crossover due to the poor ion selectivity of membranes could be largely neglected. The concept of ‘unbalanced’ was introduced to specify the capacity-limiting side and non-capacity-limiting side, making sure that the capacity decay was directly related to the capacity limiting side as a result of decomposition of the redox-active species. All the symmetric cell tests in this work employed anion exchange membrane FAPQ-350 (Fumatech GmbH) as the separator because the investigated hydroquinones were positively charged in acidic solution. The cell setup was connected to a peristatic pump (Heidolph pumpdrive 5101) with a flow rate of 16 mL·min^−1^. The prepared 50% SOC solution was put on both reservoirs with a volume difference of 1~2 mL. The cathode side was taken as the non-capacity-limiting side (NCLS) and the anode side as the capacity-limiting side (CLS). The charge/discharge program refers to the literature [8,30,33] with necessary modifications on the conditions. The symmetric cells of **O-1**, **O-3** and **O-5** were investigated using both the potentiostatic (PS) cycling method and the galvanostatic (GS) cycling method. In the PS cycling, the charge–discharge voltage was set at 0.3/−0.3 V and the cutoff current was 3/−3 mA, with ten seconds rest when switching between charge and discharge (Figure 6a). In the GS cycling, the cutoff voltage was set at 0.3/−0.3 V. Current varied according to the theoretical capacity and measured cell resistance (Table A1 in Appendix A).

## 5. Conclusions

A series of four hydroquinone derivatives substituted with morpholinomethylene and/or methyl groups were successfully synthesized via a one-step Mannich reaction. All structures were subsequently confirmed by ^1^H NMR, ^13^C NMR, HPLC and MS. The rapid synthesis promises easy scale-up and hence good competitiveness for lowering the cost of RFB systems in practical applications. The stability of the four molecules (**O-1**, **O-3**, **O-5** and **asym-O-5**) was evaluated by symmetric flow cell cycling tests in different supporting electrolytes, employing both the potentiostatic (PS) and galvanostatic (GS) cycling methods. Regardless of the supporting electrolytes applied, **asym-O-5,** with all four ortho positions centrally and asymmetrically substituted by morpholinomethylene and methyl groups, was the most stable among the four molecules, the stability of which was 0.42 to 1.8%/day. **O-5**, which had exactly the same chemical composition as **asym-O-5** but with molecular central symmetry, exhibited similar stability of 0.78 to 1.1%/day. However, **O-5** was not suitable for long-term application because its solubility in H_2_SO_4_ changed gradually due to the formation of more-stable but less-soluble polymorphs. **O-1**, which was also fully substituted with morpholinomethylene and three methyl groups, ranked the third in stability (2.9~7.3%/day). Results showed that the hydroquinone (**O-3**) without two unoccupied protons was highly unstable in 1 M H_3_PO_4_ and displayed a capacity decay rate of 48.5 to 52.7%/day. This fast capacity fade stemmed from decomposition reactions forming insoluble 2,5-dioxoterephthalic acid. Clearly, protecting all ortho position of quinone derivatives is essential for introducing a chemically stable compound as the catholyte in ORFBs. The differences in stability of the three fully substituted hydroquinones provokes the need to study how different substitution patterns can affect molecular stability, thereby providing a future guide for molecular design targeting high stability.

Although the three fully substituted hydroquinones represent difference in cycling stability, they are all well capable of accessing high shares of theoretical capacity (82 to 97%), significantly higher than AQDS derivatives. Further investigations on the dimerization behavior of this series of hydroquinones will certainly provide valuable insight to realize two-electron transfer at higher concentrations. As compared to reported redox-active organic molecules, **asym-O-5** is a good candidate for flow batteries.

## Figures and Tables

**Table 1:** Symmetric cell cycling tests of **O-1**, **O-3**, **O-5** and **asym-O-5,** using the PS and GS cycling methods.

Condition	Method	Cell Resistance/Ω	Capacity Decay Rate (Cycle)
**O-1**: 0.05M, 1 M H_2_SO_4_	GS	0.135/0.120	2.9%/day, 0.072%/cycle (378)
**O-1**: 0.1 M, 1 M H_2_SO_4_	PS	0.175/0.116	5.7%/day, 0.117%/cycle (276)
**O-3**: 0.1 M, 1 M H_3_PO_4_	GS	0.456/0.357	48.5%/day, 0.43%/cycle (221)
**O-3**: 0.1 M, 1 M H_3_PO_4_	PS	0.380/0.346	52.7%/day, 0.235%/cycle (300)
**O-5**: 0.075 M, 1 M H_2_SO_4_	GS	0.169/0.160	1.1%/day, 0.069%/cycle (179)
**O-5**: 0.05 M, 1 M H_2_SO_4_	PS	0.155/0.125	0.78%/day, 0.056%/cycle (253)
**Asym-O-5**: 0.1 M, 1 M H_2_SO_4_	GS	0.134/0.122	1.8%/day, 0.204%/cycle (179)
**Asym-O-5**: 0.1 M, 1 M H_2_SO_4_	PS	0.144/0.128	0.46%/day, 0.042%/cycle (309)

**Table 2:** Accessible capacity of the four hydroquinones.

Catholyte	Anolyte	Accessible Capacity
0.1 M **O-1** in 1 M H_3_PO_4_	0.1 M **O-1** in 1 M H_3_PO_4_	96%
0.1 M **O-3** in 1 M H_3_PO_4_	0.1 M **O-3** in 1 M H_3_PO_4_	20%
0.1 M **O-5** in 1 M H_3_PO_4_	0.1 M **O-5** in 1 M H_3_PO_4_	85%
0.1 M **asym-O-5** in 1 M H_3_PO_4_	0.1 M **asym-O-5** in 1 M H_3_PO_4_	90%
0.1 M **O-1** in 1 M H_2_SO_4_	0.1 M **O-1** in 1 M H_2_SO_4_	97%
0.05 M **O-5** in 1 M H_2_SO_4_	0.05 M **O-5** in 1 M H_2_SO_4_	88%
0.1 M **asym-O-5** in 1 M H_2_SO_4_	0.1 M **asym-O-5** in 1 M H_2_SO_4_	82%

**Table 3:** Summary of the stability of studied hydroquinones.

Molecule	Capacity Decay Rate
**O-1**	2.9 to 7.3%/day
**O-3**	48.5 to 52.7%/day
**O-5**	0.78 to 1.1%/day
**asym-O-5**	0.45 to 1.8%/day

**Table 4:** Accessible capacity of quinone-based flow batteries in the literature.

Reference	Electrolytes	Accessible Capacity
Li et al. [37]	0.1 M BQDS ^a^/0.1 M 2,7-AQDS in 1 M H_2_SO_4_	65% ^b^
Zhang, Li and Chu [38]	0.05 M BQDS/0.05 M ARS ^c^ in 1 M H_2_SO_4_	42%
Cao et al. [39]	1 M BQDS/1 M 2,7-AQDS in 1 M H_2_SO_4_	26% ^b^
Yang et al. [40]	1 M BQDS/1 M 2,6-AQDS in 1 M H_2_SO_4_	35%

^a^: BQDS: 1,2-dihydrobenzoquinone-3,5-disulfonic acid; ^b^: adapted from Table 1 presented by C. Wiberg et al. [25]; ^c^: ARS: 3,4-dihydroxy-9,10-anthraquinone-2-sulfonic acid.

## Data Availability

The data presented in this study are available on request from the corresponding author.

## Sample Availability

Samples of the compounds **O-1**, **O-3**, **O-5** and **asym-O-5** are available from the authors.

## Appendix A

**Table A1:** Condition, charge/discharge current and cell resistance of the symmetric cell cycling tests using the GS cycling method.

Condition	Current (mA)	Cell Resistance (Ω)
19 mL 0.05 M **O-1** in 1 M H_2_SO_4_	50	0.135/0.120
9 mL 0.1 M **O-3** in 1 M H_3_PO_4_	30	0.456/0.357
7 mL 0.075 M **O-5** in 1 M H_2_SO_4_	20	0.169/0.160
9 mL 0.1 M **asym-O-5** in 1 M H_2_SO_4_	20	0.134/0.122
